# Personal

**Published:** 1901-12

**Authors:** 


### PERSONAL.

DR. N. W. WILSON, of Buffalo, has been given the honorary degree of medicine man by the Broule Sioux Indians whom he served as such at the Pan-American Exposition. There was great ceremony at the council house when the title of He-wakes-them-up was conferred with the medical degree. The giving of the name refers to the illness of Chief Blue Horse's wife. The old squaw fainted one day in July and Dr. Wilson revived her. Indians think persons die when they faint and look upon their revival as somewhat of a miracle.

---

MR. "DOC." WADDELL, the efficient and courteous press agent of the Indian Congress of the Pan-American Exposition, contributed greatly to its popularity. He is an experienced newspaper man having served in all capacities from that of "devil" to the city editor of a large newspaper. His experience is such in the show business, and it being so much to his liking, that it is more than probable he will be heard from again in that field. The JOURNAL wishes him the success he so justly deserves in whatever undertaking he may embark.

---

DR. T. GAILLARD THOMAS, of New York, who attained his seventieth birthday, November 21, 1901, was tendered a complimentary dinner in celebration thereof at Sherry's, which was attended by a large number of professional friends of the distinguished physician.

---

DR. CATHERINE E. KELLY, U. of B. '99, was interne at the Children's Hospital for eighteen months after graduation. Dr. Kelly has established an office at 585 Fillmore Avenue, Buffalo. Her residence is at 45 Highland Avenue, as heretofore.

---

DR. EARL G. DANSER, of Buffalo, was elected to the office of coroner at the last election. This is a gratifying fact to the medical profession, which has long contended that a competent physician should always be chosen to this office.

---

DR. L. DUNCAN BULKLEY, of New York, removed November 1 to 531 Madison Avenue, entrance on 54th Street, New York City. Consultation hours, 9 to 1; also Monday and Thursday from 4 to 6 p.m.

---

DR. LORENZO BURROWS, JR., of Buffalo, has been appointed ophthalmic examiner for the New York State School for the Blind at Batavia. This is an excellent selection.

DR. CHARLES O. CHESTER, of Buffalo, has removed from 201 to 217 Franklin Street, where he will continue the practice of laryngology.

